# Application of Removable Wrist Splint in the Management of Distal Forearm Torus Fractures

**DOI:** 10.5812/traumamon.5094

**Published:** 2013-01-15

**Authors:** Mahmood Karimi Mobarakeh, Ali Nemati, Reza Noktesanj, Amirhossein Fallahi, Saeed Safari

**Affiliations:** 1 Department of Orthopedic and Trauma Surgery, Kerman University of Medical Sciences, Shahid Bahonar Hospital, Kerman, IR Iran; 2 Department of Emergency Medicine, Shahid Beheshti Medical University, Imam Hossein Hospital, Tehran, IR Iran

**Keywords:** Fractures, Bone, Forearm, Wrist Joint, Pediatrics

## Abstract

**Background:**

There is considerable variation in the treatment of distal forearm torus fractures (DFTF), from soft bandaging to cast immobilization.

**Objectives:**

The present study aimed to show the result of removable wrist splint (RWS) in the treatment of these fractures.

**Materials and Methods:**

One hundred forty two children aged less than 17 years old with DFTF were studied prospectively. These patients were randomly treated either by a short arm cast (SAC) or a RWS for three weeks. Finally the treatment results of the two groups were compared.

**Results:**

There were no significant differences regarding degree of pain, compliance or complications between RWS and SAC groups. Resource savings can be made with this approach also patients’ and parents’ satisfaction can be increased without compromising patients' care.

**Conclusions:**

RWS can be considered as an easy and acceptable treatment modality with very low costs and complications in the management of DFTF.

## 1. Background

DFTFs are very common and they occupy a considerable part of orthopedic workload in pediatric Emergency Departments (ED). The terms “buckle” and “torus” are interchangeably used for these fractures. This means a compression failure of the bone and it normally occurs in the transitional zone between the woven metaphyseal and lamellar dense diaphyseal bone (1). It is the most common fracture of children`s forearm (2).The patients commonly present with a history of fall onto the outstretched hand, tenderness and swelling of distal forearm. Radiographs confirm the diagnosis (1). There is a considerable discrepancy in the treatment of DFTF, between different hospitals and consultants. Immobilization in a SAC for three weeks and a follow-up visit for cast removal and control radiography is the standard treatment (1, 2). The other alternatives are application of a RWS (3), lightweight backslab (4) and soft bandage (5). Recent studies have shown that immobilization in circumferential cast is unnecessary and a short period of wrist support can be enough (1).

## 2. Objectives

The main purpose of this study is to show the treatment outcomes of DFTF managed with RWS compared with the standard treatment.

## 3. Materials and Methods

**Figure 1:**
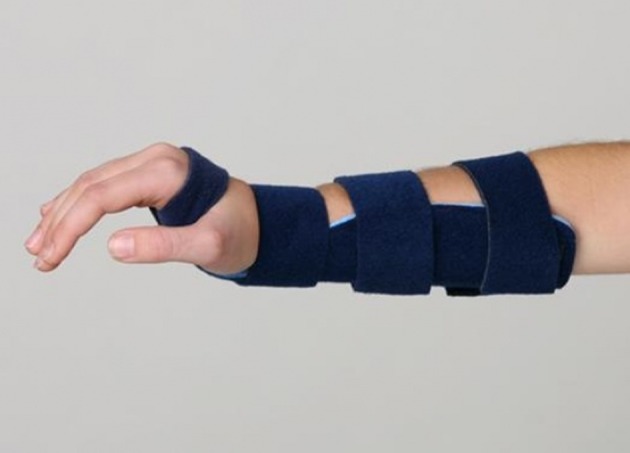
The Removable Wrist Splint (RWS) Used in the Current Study

All recognized DFTF patients, who referred to the orthopedic clinic of Shahid Bahonar Hospital (an educational hospital of Kerman province, Iran) from July to December 2010, were enrolled in this clinical trial. They were randomly divided into two groups on the day of attendance in the clinic. One group was managed with SAC and the other with RWS (figure 1). Ethics committee of Shahid Bahonar University of Medical Sciences approved the research. A full verbal and written explanation was given to the parents in RWS group. All the parents in RWS group gave an informed consent prior to being included into the study. The duration of treatment was three weeks for both groups. Appointments were made, for three weeks later, for the SAC group for cast removal, control radiography and filling the follow-up form. The patients in the RWS group were followed up by phone upon termination of their treatment period. Questions were asked to fill out the follow up form, regarding pain severity, convenience of treatment, satisfaction with the “one stop” service as well as to document any cast or splint problems or complications. Patients were asked about pain alone or other symptoms, either in the wrist splint or the plaster cast. The scale of pain assessment consisted of a semantic scale similar to an analog visual scale, but they were not exactly validated. The patient reported the results in terms of no pain, pain on activity or pain at rest. The satisfaction of patients was measured by Verhaar scale.

The parents` responses to the questionnaire were analyzed by SPSS (ver. 12) and the studies were compared using the Chi-Square test. The costs of different materials used in the two methods of treatment were supplied by the contract department.

## 4. Results

One hundred forty two DFTF children referred to the orthopedic clinic during the 6 month period of study. 103 (72.5%) patients were boys and 39 (27.5%) girls, with a mean age of 9.5 ± 1.9 years (1.2 to17). There were 114 (80.3%) isolated fractures of the radius, 2 (1.4%) isolated fractures of the ulna and 26 (18.3%) fractures of both radius and ulna. In 61 (43%) patients fracture was in the right hand, in 79 (55.6%) patients it was in the left hand and in 2 (1.4%) patients bilaterally.

65 (45.8%) patients were treated with RWS and 77 (54.2%) with a cast. One patient in the RWS group and 4 in the SAC group failed to take part in the follow-up and were excluded from the study. The most common cause of fracture was falling down in 108 (76.1%) cases, sport trauma in 25 (17.6%) cases and others 9 (6.3%). The patients attended the fracture clinic from 4 hours to 7 days after the injury (1.66 ± 1.13 days). In the SAC group at the follow-up all fractures were united clinically and radiographically with no loss of position. There were no adverse events or skin problems in either group. Patients treated with RWS removed their splint at 3.15 ± 0.75 weeks and the cast group at 3.14 ± 0.75 weeks. For those treated with RWS 28 (41.3%) cases and in the cast group 24 (31.2%) experienced mild to moderate pain with activity (P = 0.61). None of the patients had pain at rest. 58(89%) of patients in the RWS group and 66(86%) of patients in the SAC group found their treatment convenient (0.52). Compliance with both types of treatment was good except in 5 very young patients who tried to remove their splints soon after they had been applied. There was no significant difference between the two groups regarding compliance (P = 0.53). 11(7.7%) patients developed a rash under the splint. There were no cases of difficulty with removal of the wrist splint, and no residual symptoms. None of the casts broke or became soft and 5(3.5%) patients developed edema under the cast.

### 4.1. Cost-benefit Analysis

Treatment of the SAC group which involved screening visits, radiography in ED, visits to the fracture clinic, different resources used for cast application, second attendance for cast removal and radiography cost 15.3 US dollars in Iran. Treatment of the RWS involved screening visit, radiography in ED, visits in the fracture clinic and application of a wrist splint cost 9.3 US dollars in Iran.

## 5. Discussion

The results of the current study indicated that RWS can be considered as an easy and acceptable treatment modality with very low cost and complications in management of DFTF. DFTF is a fracture in which the bone cortex bulges due to a longitudinally applied compressive force. Recent research emphasizes that this fracture is a stable injury and can be treated merely by supportive care and pain control (1-4). This may account for the delayed presentation of such injuries to the ED after what has been perceived as a trivial injury by the parents. To be sure that fractures at risk of later displacement are not accidentally included, accurate diagnosis is very important and a single visit to the fracture clinic is essential. There is a variety of treatment options; from long or short arm casts to forearm backslabs, RWS and soft bandages. Plint et al, demonstrated that patients were treated with RWS had a better physical function and less difficulty with some activities compared with those treated with a cast, without any differences between their level of pain (6). Symons found that if the choice was given to both groups either to remove the backslab at home or attend the fracture clinic, parents would prefer to remove their children's backslab at home (7). In this series patients were attending the fracture clinic with a mean delay of 1.66 ± 1.13 days after the injury (minimum: 4 hours and maximum: 7 days). 65 (45.8%) patients were treated with an RWS and 77 (54.2%) with a cast. There were no significant differences regarding pain experience, compliance and complications between the two groups. Parental satisfaction with such a regimen was very high. Patients and their parents reported that they liked the fact that the RWS could be removed for bathing and many of them said that the children had removed the splint before the end of the three weeks since the pain had settled down. The work load of the plaster technicians, the number of patients attending the clinic for follow-up visit and the time spent in the department by children and careers would also have been reduced. One of the main advantages of RWS was that a child did not need to return to the fracture clinic to remove the splint. It can be easily removed by parents at home by providing full verbal and written instructions at the first visit in the fracture clinic. Farman showed that post cast studies of torus fractures were unnecessary and multiple radiographs did not change fracture management (8). All fractures united clinically and radiologically without any problem at the follow-up. There were no complications suggesting that radiological follow-up was required. It is important to consider that economic analysis provides a powerful tool to evaluate health-care technologies and treatment strategies (9). Considerable savings can be made in terms of cost and workload by discharging the patients with RWS. Treatment of DFTF by RWS will result in a total saving of 6 US dollars per case. RWS can be considered as an easy and acceptable treatment modality with very low cost and complications in the management of DFTF.
